# Supplementary material for: Knowledge and attitudes about HIV pre-exposure prophylaxis: Evidence from in-depth interviews and focus group discussions with policy makers, healthcare providers, and end-users in Lesotho
Source: PLOS Glob Public Health. 2022 Oct 3;2(10):e0000762. doi: 10.1371/journal.pgph.0000762 (PMC10021845; doi:10.1371/journal.pgph.0000762)
Supplement: Attachment — Submitted filename: PointbyPointReply_2022-06-16.docx [file pgph.0000762.s004.docx]

**Authors’ response to reviews**

**Paper ID:** PGPH-D-22-00344

**Title:** Knowledge and attitudes about HIV pre-exposure prophylaxis: Evidence from in-depth interviews and focus group discussions with policy makers, healthcare providers, and end-users in Lesotho

**Authors:** Pascal Geldsetzer, Joy J. Chebet, Tapiwa Tarumbiswa, Rosina Phate-Lesihla, Chivimbiso Maponga, Esther Mandara, Till Bärnighausen, Shannon A. McMahon

**Date:**  June 16^th^, 2022

**Author's response to reviews:** see below

Dear Dr. Monera-Penduka:

We would like to first thank the reviewers for their helpful and constructive feedback. We thank all reviewers who describe the work as technically sound, with data that support the conclusions in a rigorous manner, and with writing that is clear, correct and unambiguous. We are thankful to Reviewer 1 who noted that our paper has made an “important contribution to the field of PrEP and global public health”. We are grateful to Reviewer 3 who described our paper as being “timely for programming efforts in Lesotho and could serve as a model for conducting formative research around the same topic in other countries”. We also thank Reviewer 2 for their thoughtful feedback.

Please see below a point-by-point response to all minor and major comments from each reviewer. We hope that these changes (also highlighted via track changes in the attached manuscript) meet the expectations of reviewers and editors. We have attached an additional questionnaire related to PLOS Global Public Health’s Inclusivity in Global Research. We have also followed up with all co-authors to confirm that they have co-authored the paper. We look forward to your feedback.

Kindly,

The Authors

| **Editor** | |
| --- | --- |
| Any changes to the reference list should be mentioned in the rebuttal letter that accompanies your revised manuscript. If you need to cite a retracted article, indicate the article’s retracted status in the References list and also include a citation and full reference for the retraction notice. | More up-to-date references have been added to the manuscript. These changes are reflected in the manuscript in track changes, and also below when responding to reviewer comments. |
| The [PLOS Data policy](https://journals.plos.org/globalpublichealth/s/data-availability) requires authors to make all data underlying the findings described in their manuscript fully available without restriction, with rare exception. The data should be provided as part of the manuscript or its supporting information, or deposited to a public repository. For example, in addition to summary statistics, the data points behind means, medians and variance measures should be available. If there are restrictions on publicly sharing data—e.g. participant privacy or use of data from a third party—those must be specified. | In compliance with PLOS data policies, authors have made available the interview and focus group discussion guides and added them to the manuscripts as Appendix 1. The codebook used to code transcripts has also been added to the manuscript as Appendix 2. |
| **Reviewer 1** | |
| Thank you so much for letting me review this important contribution to the field of PrEP and global public health. This is a well written and interesting piece, which I am sure will contribute to the fight against HIV. | We thank the reviewer for highlighting that this work is well written and interesting. |
| **Sampling**. I have a comment regarding the sampling. The authors write that they achieved diversity among PrEP end users to represent a variety of ages etc. When I am looking into Table 1, I notice that there are important gaps in representation and this needs to be addressed in the text. Females were 69% of the users, 86% of former users and 100% of the decliners (no non-binary or other trans people?). | The reviewer raises a valid point. Our sampling strategy did not intentionally exclude individuals identifying as male, non-binary or transgender. As noted in the manuscript, however, the majority (79%) of the end-users identified as female. A recommendation for more inclusive research in the future has been incorporated into the limitations section with the following text:  *Finally, although this study included respondents across several ages, we encourage future research that more pointedly captures perspectives from those who identify as male or non-binary; men retain tremendous influence on household decision making related to healthcare seeking and their insights thus merit pointed examination. Furthermore, men who have sex with men bear a disproportionate burden of risk relative to HIV. We hope our findings spark future research among these groups.* |
| **Male exclusion.** Where are the men? If the aim is to increase uptake, one has to understand the groups who currently are not being reached. Men who have sex with men were mentioned once but looking at the Respondent HIV Risk category, there were none included? This is problematic for several reasons, the main being that MSM and MSM sex workers are usually disproportionate negatively affected by HIV also in settings with a high prevalence in the general population. Although homosexuality is not criminalised in Lesotho, there is no legal protection and stigma remains high, this is a barrier towards getting MSM/MSM sex workers into clinics for PrEP enrollments. Under “PrEP knowledge” I read that end-users cognizant that PrEP users were often MSM. At the same time, they are not part of this study? This must be discussed in Limitations. | The reviewer’s point is well-taken. This insight has been incorporated into the limitations section with the following text:  *Finally, although this study included respondents across several ages, we encourage future research that more pointedly captures perspectives from those who identify as male or non-binary; men retain tremendous influence on household decision making related to healthcare seeking and their insights thus merit pointed examination. Furthermore, men who have sex with men bear a disproportionate burden of risk relative to HIV. We hope our findings spark future research among these groups.* |
| **Former users.** Regarding the large group of former users, I would like to know why they stopped? They had positive attitudes towards PrEP but chose not to use it any more. New life choices? Desirability bias? A large group of former users had the experience of sex work. I wonder, did they stop sex work or why did they stop? | Behavioral patterns related to PrEP use, including reasons for decline, represents an exciting vein of research, but was not the focus of this study (which is rather focused on knowledge, attitudes and misconceptions related to PrEP). |
| **U=U in the context of PrEP**. The respondents talk about how PrEP enables relationships with people living with HIV and that is great. But doesn’t U=U do that as well? I would like a short paragraph on that. | Following the reviewer’s feedback, the following text has been added to incorporate discussions around U=U:  *Furthermore, great care is required in distinguishing conceptual differences between PrEP as a prophylactic drug for use by an HIV negative individual, and the reduced risk of HIV transmission resulting from consistent ART use in persons living with HIV. Previous studies have demonstrated viral load is associated with HIV transmission, with the risk of transmission diminishing with lower viral levels (new citations: Cohen et al 2016; and Roger et al 2019). This concept showing undetectable levels of HIV equaling untransmissibility of the virus (undetectable equals untransmittable; U=U) requires the HIV positive individual to achieve viral suppression, which may take over 6 months of consistent ART use (new citations: Ridell et al 2018). While both strategies — PrEP and U=U — are aimed at reducing risk of HIV transmission, both require adequate counseling and monitoring by health providers.* |
| Also, in my own previous research on PrEP**, being on PrEP meant being an ally to the community of people living with HIV/AIDS.** Did someone voice that? I noted the opposite, that the mix-up between ART and PrEP caused stigma. | While this is an interesting perspective raised in other work, respondents in this study did not raise the issue of allyship.  In line with the reviewer’s previous research, respondents in this study also discussed the conflation between ART and PrEP. We discuss this in the discussion:  *In addition to the misconception that PrEP protects against STIs other than HIV and pregnancy, our respondents reported a common conflation of ART and PrEP. Addressing this misconception is important because it may lead to increased stigma around the use of PrEP, which in turn may negatively affect PrEP use and disclosure (18,29,41). In some studies, stigma has led to early discontinuation of PrEP use (29). Ways to more clearly distinguish PrEP from ART could include packaging that bears no semblance to ART – both in the packet and the pill appearance itself (19). Additionally, tailored marketing terminology, adapted to the target audience and delivered in accessible ways, could highlight the differences between ART and PrEP.* |
| **Negative aspects or reservations about PrEP**. As I mentioned earlier, if the desired implication is a deeper understanding of how to facilitate a broader appeal of PrEP programs, it would have been interesting to learn more about negative aspects or reservations about PrEP. **It is mentioned briefly in the last paragraphs of the Results section but I would like to see a little bit more on that.** Are there obsticles articulated from a structural perspective, for instance faith-based organisation or similar? | While the reviewer raises an important and interesting point, discussion around structural and other obstacles of initiating PrEP was beyond the scope of this paper. Here we solely report findings on the knowledge, attitudes and perceptions of PrEP. |
| **Reference outdated.** I noted that the reference on the efficiency of PrEP is, when writing about PrEP, a bit old (2016). Maybe a newer reference, with the focus on real-life settings. | The reviewer’s feedback is well-taken. The following more recent systematic reviews and meta-analyses summarizing the efficacy of PrEP have been added to the manuscript:   1. Pilkington V, Hill A, Hughes S, Nwokolo N, Pozniak A. How safe is TDF/FTC as PrEP? A systematic review and meta-analysis of the risk of adverse events in 13 randomised trials of PrEP. J Virus Erad. 2018 Oct 1;4(4):215-224. PMID: 30515300; PMCID: PMC6248833. 2. Riddell, J., 4th, Amico, K. R., & Mayer, K. H. (2018). HIV Preexposure Prophylaxis: A Review. *JAMA*, *319*(12), 1261–1268. <https://doi.org/10.1001/jama.2018.1917> 3. Powell, V. E., Gibas, K. M., DuBow, J., & Krakower, D. S. (2019). Update on HIV Preexposure Prophylaxis: Effectiveness, Drug Resistance, and Risk Compensation. *Current infectious disease reports*, *21*(8), 28. <https://doi.org/10.1007/s11908-019-0685-6> |
| **Clarity.** Under the headline “PrEP knowledge”, the first paragraph and quotation. I am not sure how to understand the text and how the quotation underpin the text. | This text has been modified for clarity. |
| **Reviewer 2.** | |
| Abstract: Clear and easy to comprehend. Although this evidence: what evidence? | This text has been modified for clarity. |
| Intro: Prevalence data from 2018 used. | This text has been updated to reflect the UNAIDS factsheet 2020 |
| Study design: Would be nice to have added a sentence mentioning that the study was qualitative in nature. | The qualitative nature of the study has been added into the second paragraph of the methods section.  *In this qualitative study, we conducted semi-structured in-depth interviews (IDIs) with policy makers and implementing partners* |
| **Limitations:** Through discussions with the Lesotho Ministry of Health: Key informants were identified in collaboration with the Ministry of Health: Doesn’t it introduce biasness- how was it mitigated? | In response to the reviewer’s comment, we have edited the sampling text to reflect the inclusion of snowball sampling as a means to expand our pool beyond those initially identified via MoH officials. Additionally, we have added the following text to the limitations section of the discussion:  *Confidentiality was particularly salient for policy makers and implementing partners, who were identified in collaboration with Ministry of Health officials. Given the high-ranking positions policy makers and implementing partners included in the study occupied, and that this work was being done, in part, to support the implementation of the national PrEP program, it is unlikely that the involvement of Ministry of Health officials hindered the respondents’ full engagement in the interviews.* |
| **Data analysis:** Themes were developed from detailed debriefing notes and analysis workshops conducted between interviewers, FGD moderators, and the study investigators: Who facilitated the analysis workshops? How many workshops were required? How were different themes from different individuals harmonized or agreed? | This text has been modified for clarity.  *Following each day of data collection, interviewers and FGD moderators debriefed with a lead investigator (JJC) (23). In total, more than 11 debriefing sessions were undertaken, and these sessions served as an opportunity to organize data, and identify emerging themes as well as areas of additional inquiry and saturated topics.*  Additionally, the following text discussing the codebook has been added:  *We then further teased out these themes via line-by-line coding, with all codes being entered into a codebook and applied to all transcripts in Atlas.ti. (22). The full codebook with the emerging themes (n=3) and codes (n=15) is available in Appendix 2.* |
| **Data analysis.** If the themes were developed from the briefing notes- how were interview transcriptions used for the themes identification?  How many key themes were generated? | This text has been modified for clarity.  The full codebook with codes listed below themes is available in Appendix 2. |
| **Results:** Prep-knowledge:  End-users were cognizant that PrEP is an antiretroviral drug (ARV) and about the priority groups for PrEP use, including individuals in serodiscordant relationships, individuals with multiple sexual partners, and MSM: Sentence difficult to comprehend | This text has been modified for clarity.  *End-users described a generally high-level of understanding in terms what PrEP is made of, how it is used and who is eligible for PrEP. End-users described PrEP is an antiretroviral drug (ARV), named priority groups for PrEP use as, generally, individuals in serodiscordant relationships, individuals with multiple sexual partners, and MSM.* |
| **Quantifying qual data:**  Would be nice to quantify data on end-users like - overwhelming number of people heard about prep from radio and television: - to a lesser degree - a minority of  While the researcher have provided numbers (two respondents, one users etc) in many instances. | Thank you for raising this valid point. Our reasoning for not consistently stating the numbers of respondents that talk about a particular theme is that although we see a frequency of themes, we cannot state that a given number of individuals definitively hold that view. Due to the semi-structured nature of our interview guide and the iterative nature of qualitative research generally, no two questions were asked in exactly the same way, nor answered in exactly the same way. We therefore prefer to present a general overview, rather than quantifying qualitative insights. |
| **Discussion:** Among policy makers, implementing partners, and healthcare providers, PrEP evoked a sense of hope for addressing the HIV epidemic in Lesotho: Just curious if the epidemiological data supported this since PreP was initiated in 2016 (almost 5 years ago) in 5 of 10 districts | Following the reviewer’s query, the authors conducted a PubMed search of publications on the subject. We were unable to find population-level epidemiological studies discussing the impact of PrEP on HIV prevalence since PrEP’s introduction in 2016 |
| It would be nice to have information on the need of further studies in the recommendation, since there are few studies as mentioned in the introduction. | To highlight the need for further studies, the following text has been added to the discussion section of the manuscript:  *In expanding the PrEP program nationally to the general population in Lesotho, concerted sensitization efforts and community mobilization are, therefore, likely to be important to generate demand for PrEP. Furthermore, context-specific research on the level of PrEP knowledge among lay Basotho people would be useful in the scale-up and longterm implementation of a national PrEP program.*  *We, therefore, recommend public health communication campaigns targeted specifically at addressing the misconceptions found in this study — that PrEP protects against STIs and pregnancy, PrEP causes seroconversion and one dose of the drug offers lifelong protection from HIV infection — to support a successful PrEP delivery strategy in Lesotho and similar settings.*  In reference to inclusion of participants identifying as male, we have added:  *…although this study included respondents across several ages, we encourage future research that more pointedly captures perspectives from those who identify as male or non-binary; men retain tremendous influence on household decision making related to healthcare seeking and their insights thus merit pointed examination. Furthermore, men who have sex with men bear a disproportionate burden of risk relative to HIV. We hope our findings spark future research among these groups.* |
| Would be nice if the recommendation also provide more on the communication strategy details like behavior change or campaign, targeted messaging to youth, women etc- if its relevant to the study findings. | The following recommendation around a communication campaign has been added to the manuscript:  *We, therefore, recommend public health communication campaigns targeted specifically at addressing the misconceptions found in this study — that PrEP protects against STIs and pregnancy, PrEP causes seroconversion and one dose of the drug offers lifelong protection from HIV infection — to support a successful PrEP delivery strategy in Lesotho and similar settings.* |
| **Reviewer 3.** | |
| This manuscript describes results from qualitative research on Knowledge and attitudes about HIV pre-exposure prophylaxis with policy makers, healthcare providers, and end-users in Lesotho. The manuscript is timely for programming efforts in Lesotho and could serve as a model for conducting formative research around the same topic in other countries. | We thank the reviewer for highlighting that our work is timely and could serve as a model for related formative research. |
| Generally, the manuscript is well-written and clear in both the methods and discussion. A few comments are noted:  Abstract Nice summary of the results. | We thank the reviewer for highlighting that our work is well written. |
| Introduction The authors state that “Research in sub-Saharan Africa done in this area has mostly been carried out in the context of large-scale clinical trials (18–20). There is, thus, little evidence on awareness, knowledge, and perceptions of PrEP from effectiveness and implementation studies conducted in sub-Saharan Africa.” Yet they cite a multitude of studies (references 23-25, 40, 43) from the region in their discussion. I am not clear the point the authors are trying to make in the introduction. | Clarification on the need of additional evidence in around PrEP knowledge and awareness has been added:  *There is, thus, need for additional evidence on PrEP awareness, knowledge, and perceptions from effectiveness and implementation studies conducted as part of large-scale PrEP programming as more sub-Saharan African countries incorporate PrEP into their respective national HIV/AIDS control strategies.* |
| **Methods** Were all PrEP end-users adults? Perhaps it would be useful to state the eligibility criteria for participation a bit more clearly. | The reviewer raises a valid ethics-related point. All those who took part in this study were, in-fact adults. This has been clarified in the text and can also be see in table 2 which lists the participant’s age ranges.  *We conducted in-depth semi-structured interviews with adult PrEP end-users – current and former users, and decliners – to elicit detailed narratives and firsthand accounts of individual experience with PrEP.* |
| The authors state that “facilities were selected for the recruitment of study participants based on: 1) type of health facility: including government, private and faith-based facilities; 2) mode of PrEP delivery: community- and facility based; and 3) location: urban and rural.” It would be interesting to have this information reflected in Table 1 as well as the geographic spread of all respondents. | The reviewer raises a valid point. While these factors were used in the selection of study sites, they were not included as variables for analysis. We have, however, included if the interviews/focus group discussions were conducted in an urban or rural site in table 1. |
| Also in Table 1, “number of data collection activities” is a bit strange in terms of a header – perhaps revise? | This has been edited  *Table 1. Number of semi-structured in-depth interviews and focus group discussions conducted* |
| What is the difference between IDIs and semi-structured IDIs? In the narrative, the authors state that all IDIs are semi-structured. | We thank the reviewer for noting this. All the interviews conducted were semi-structured. Table 1 and related text has been edited to clarify this point.  *All IDIs and FGDs were semi-structured. While the research team developed a list of pertinent questions for each respondent group, research assistants were at liberty to alter the order and sequence of research questions based on the flow of the interview or discussion (see appendix 1 - interview and discussion guides).* |
| Results No comments; very nice reporting of qualitative results | We thank the reviewer for highlighting that our work represents a nice reporting of qualitative results. |
| Discussion Discussion is very clear and organized. | We thank the reviewer for highlighting that our discussion is clear and organized. |
| Additional messaging to consider would be clear articulation of the need for dual protection if not interested in getting pregnant, and clear messaging about male condom use (i.e, not needing to use condoms for disease prevention if using PrEP; need to use condoms or other contraceptive method to protect against unintended pregnancy). | The following recommendation around a communication campaign has been added to the manuscript:  *We, therefore, recommend public health communication campaigns targeted specifically at addressing the misconceptions found in this study — that PrEP protects against STIs and pregnancy, PrEP causes seroconversion and that one dose of the drug offers lifelong protection from HIV infection — to support a successful PrEP delivery strategy in Lesotho and similar settings.* |
